# Validity of a questionnaire-based diagnosis of chronic obstructive pulmonary disease in a general population-based study

**DOI:** 10.1186/1471-2466-14-49

**Published:** 2014-03-21

**Authors:** Nicola Murgia, Jonas Brisman, Annika Claesson, Giacomo Muzi, Anna-Carin Olin, Kjell Torén

**Affiliations:** 1 Department of Occupational and Environmental Medicine, Sahlgrenska University Hospital, University of Göteborg, Göteborg, Sweden; 2 Section of Occupational Medicine, Respiratory Diseases and Toxicology, University of Perugia, Perugia, Italy

**Keywords:** Airway obstruction, Spirometry, Sensitivity, Accuracy, ATS/ERS, GOLD

## Abstract

**Background:**

The diagnosis of chronic obstructive pulmonary disease (COPD) is based on airflow obstruction. In epidemiological studies, spirometric data have often been lacking and researchers have had to rely almost solely on questionnaire answers. The aim of this study is to assess the diagnostic accuracy of questionnaire answers to detect COPD.

**Methods:**

A sample of the Swedish general population without physician-diagnosed asthma was randomly selected and interviewed using a respiratory questionnaire. All eligible subjects aged 25–75 years (n = 3892) performed spirometry for detection of airflow obstruction using Global Initiative for Chronic Obstructive Lung Disease (GOLD) or American Thoracic Society (ATS)/European Respiratory Society (ERS) criteria. Sensitivity, specificity, positive likelihood ratio (LR+), positive predictive values (PPVs), and negative predictive values (NPVs) were calculated to define diagnostic accuracy of questionnaire answers.

**Results:**

The sensitivity of the question “Have you been diagnosed by a physician as having COPD or emphysema?” in detecting airflow obstruction was 5.7% using GOLD, and 9.8% using ATS/ERS, criteria; specificity was 99.7% for GOLD and 99.5% for ATS/ERS. Sensitivity, specificity, and PPV were higher for the question compared to self-reported symptoms of chronic bronchitis in identifying subjects with airflow obstruction.

**Conclusions:**

The high specificity and good PPV suggest that the question “Have you been diagnosed by a physician as having COPD or emphysema?” is more likely to identify those who do not have airflow obstruction, whereas the low sensitivity of this question could underestimate the real burden of COPD in the general population.

## Background

Chronic obstructive lung disease (COPD) continues to be an important source of morbidity and mortality and a socioeconomic burden worldwide despite the attention paid by the scientific community and the availability of guidelines and recommendations on prevention, diagnosis, and treatment [1,2]. Unfortunately, there is still no consensus on the criteria for diagnosis of COPD. Nonetheless, airflow obstruction is commonly recognized as a key feature of COPD, and spirometry is the routine procedure of choice for detecting airflow obstruction and diagnosis of COPD [2,3].

The degree of airway obstruction that characterizes COPD is still under discussion since the Global Initiative for Chronic Obstructive Lung Disease (GOLD) has proposed a fixed post-bronchodilator ratio of forced expiratory volume in 1 second (FEV_1_) to forced vital capacity (FVC) of <0.70 [4] as its criterion. This is widely applied, also for its practicability, but has been criticized for the risk of overdiagnosis [5]. Other authors, on behalf of the American Thoracic Society (ATS) and the European Respiratory Society (ERS), have defined airway obstruction as a reduction in FEV_1_/FVC below the age-, gender-, and race-adjusted fifth percentile of a healthy, never-smoking population, which is regarded as the lower limit of normal (LLN) (FEV_1_/FVC/LLN) [6]. Use of this definition reduces the risk of overdiagnosing the disease, especially in elderly subjects [7].

According to the GOLD definition, even if dyspnoea, chronic cough, and chronic sputum production are often associated with COPD, the definition of COPD does not include the terms “chronic bronchitis” or “emphysema” [2,4]. In epidemiological studies, especially in follow-up, questionnaire-based surveys, or in large, population-based investigations, spirometric data are often lacking and researchers have to rely almost solely on subjects’ answers. As the questions were originally designed for detecting asthma [8,9], validity of questionnaires in detecting COPD needs to be checked against a diagnostic gold standard for the disease. Although agreement still needs to be reached on a definition of airway obstruction criteria for COPD, some researchers have tried to validate specific questionnaires based on symptoms and other variables in high risk populations (smokers). They found that an association of several questions, coupled with a scoring system, could yield reasonable sensitivity and specificity to detect airflow obstruction in these high risk groups [10,11]. In another study, out of a small sample of nurses reporting physician-diagnosed COPD, 27% had airflow obstruction [12].

The objective of this study is to assess, in a large sample of the general population, the validity of questionnaire answers to detect COPD, compared to current definitions of COPD, based on airflow obstruction.

## Methods

### Population and questionnaire

A general population sample of 6685 men and women 25–75 years old was randomly selected from the population register in Göteborg, Sweden, and sent a postal questionnaire and an invitation to undergo a clinical examination, as previously described [13,14]. The study population was recruited in 2001. The key question about physician-diagnosed COPD was introduced in 2004 and the present study was completed in 2008. Altogether 4520 men and women answered the questionnaire. An affirmative answer to the question “Have you been diagnosed by a physician as having COPD or emphysema?” defines the doctor-diagnosed COPD category. Respondents reporting cough and phlegm for at least 3 months within 1 year for 2 consecutive years fell into the chronic bronchitis category. The questionnaire also included questions about age, sex, height, weight, smoking habits, current respiratory therapy, respiratory symptoms, respiratory infections in the last year, and the question “Have you been diagnosed by a physician as having asthma?” Respondents (n = 315) with physician-diagnosed asthma were excluded, as well the 21 respondents not answering the question regarding asthma. Another 292 respondents were excluded due to missing information about spirometry and smoking habits, yielding a final number of 3892 study subjects. The study was approved by the Ethical Committee of Göteborg University (No. 237/2000) and all subjects gave their informed consent.

### Spirometry

Before the spirometry, subjects were weighed and height was measured with subjects barefoot and wearing light clothes. Spirometry was performed with a dry wedge spirometer (Vitalograph; Buckingham, UK). Forced expiratory volume in 1 second and FVC were expressed in liters. Percentages of predicted values of lung function variables (i.e., FEV_1_, and FEV_1_/FVC ratio) were calculated using the European Community for Steel and Coal (ECSC)/ERS equation [15]. No broncho-reversibility test with short-acting bronchodilators was performed. A FEV_1_/FVC ratio <0.7 indicates airway obstruction according to GOLD criteria [4,16] and a FEV_1_/FVC <1.645 × residual standard deviation (RSD) below the predicted value was used as estimation of the LLN, which is the criterion used by the ATS/ERS for defining airway obstruction [6,15].

### Statistical analysis

Continuous data are expressed as mean ± standard deviation (SD), whereas categorical data are presented as numbers and percentages. We used sensitivity, specificity, positive likelihood ratio (LR+), positive predictive values (PPVs), and negative predictive values (NPVs) to define the diagnostic accuracy of the questions, indicating self-reported, physician-diagnosed COPD and calculated for each COPD definition (GOLD and LLN-based) with 95% confidence intervals (CIs). We also assessed accuracy parameters in a sample of the population aged >40 years, in subjects not reporting wheezing, and in subcategories by gender and smoking habits (never-smokers, former smokers, current smokers). All calculations were performed with SPSS 18.0 (IBM Corp., New York, NY, USA) and Simple Interactive Statistical Analysis (SISA) free software [17].

## Results

In the whole study population, 366 subjects (9.4%) had COPD according to the GOLD criteria, whereas 163 (4.2%) had COPD according to LLN criteria (Table 1). Altogether 33 subjects (0.8%) reported doctor-diagnosed COPD, in 19 of whom (57.6%) the diagnosis was made after 2001. Ninety subjects (2.3%) were classified as having chronic bronchitis. Other characteristics of the study population, stratified for COPD diagnosis, are displayed in Table 1. Sensitivity, specificity, LR+, PPVs, and NPVs in the overall population can be seen in Table 2 (see Table 2 file). Sensitivity of the question “Have you been diagnosed by a physician as having COPD or emphysema?” in detecting airflow obstruction was 5.7% using the GOLD definition and 9.8% using the ATS/ERS definition, whereas specificity was high for both definitions, 99.7% for GOLD and 99.5% for ATS/ERS. Sensitivity, specificity, and PPV for the above question were higher than the self-reported symptoms of chronic bronchitis in identifying subjects with COPD for both airway obstruction definitions (GOLD and LLN). We performed the analysis in individuals >40 years old and in those who did not report wheezing, but the results were similar (data not shown). In Table 3, we report diagnostic accuracy data for women and men (see Table 3 file). Tables 4 and 5 describe accuracy data in never-smokers, former smokers, and current smokers for the two categories (doctor-diagnosed COPD and chronic bronchitis) (see Tables 4, 5 file). The sensitivity of the question “Have you been diagnosed by a physician as having COPD or emphysema?” was higher in smokers, compared to never-smokers (Table 4) (see Tables 4, 5 file).

**Table 1:** Characteristics of the study population

	**FEV**_**1**_/FVC <0.7 (GOLD) n = 366*	**FEV**_**1**_/FVC <1.645 SD below predicted (ATS/ERS) n = 163*	**All N = 3,892**
Physician-diagnosed COPD,%	5.7	9.8	0.8
Chronic bronchitis symptoms,%	4.6	7.4	2.3
Women,%	49.2	66.9	52.5
Age (mean ± SD), yrs	58.3 ± 9.5	54.9 ± 10.3	51.7 ± 10.6
BMI (mean ± SD), kg/m^2^	25.6 ± 3.9	25 ± 3.8	26.1 ± 4
Spirometry			
FEV_1_ (L) (mean ± SD)	2.7 ± 0.8	2.5 ± 0.8	3.3 ± 0.8
FEV_1_% (mean ± SD)	65.1 ± 5.3	61.9 ± 6.3	78.0 ± 6.3
FEV_1_/FVC (mean ± SD)	64.9 ± 5.3	61.7 ± 6.3	77.9 ± 6.4
Dyspnea,%	6.3	9.2	6.3
Wheezing,%	24.6	31.3	14.6
Treatment with respiratory drugs,%	4.6	8	1.1
Smoking habits			
Never-smokers,%	29.8	27.6	47.7
Former smokers,%	38.8	35.6	36.2
Current smokers,%	31.4	36.8	16.1
Pack/years in current and former smokers (mean ± SD)	22.6 ***±*** 15.5	22.2 ***±*** 13.8	15 ***±*** 12.2

*In the whole study population, 366 subjects (9.4%) had COPD according to the GOLD criteria; 163 (4.2%) had COPD according to LLN criteria.

ATS = American Thoracic Society; BMI = body mass index; COPD = chronic obstructive pulmonary disease; ERS = European Respiratory Society; FEV_1_ = forced expiratory volume in 1 second; FEV_1_% =% of FEV_1_ compared to the predicted value; FEV_1_/FVC = FEV_1_/forced vital capacity ratio; GOLD = Global Initiative for Chronic Obstructive Lung Disease; SD = standard deviation.

**Table 2:** Diagnostic accuracy of the question “Have you been diagnosed by a physician as having COPD or emphysema?” and of self-reported, questionnaire-based chronic bronchitis symptoms to detect chronic obstructive pulmonary disease (COPD)

		**FEV**_**1**_**/FVC <0.7 (GOLD)**		**FEV**_**1**_**/FVC <1.645 SD below predicted (ATS/ERS)**	
		**Value**	**95% CI**	**Value**	**95% CI**
Physician-diagnosed COPD	Sensitivity	0.057	0.027–0.088	0.098	0.04–0.156
	Specificity	0.997	0.994–0.999	0.995	0.993–0.998
	LR+	16.859	6.894–41.228	21.532	9.226–50.25
	PPV	0.636	0.427–0.846	0.485	0.267–0.702
	NPV	0.911	0.899–0.922	0.962	0.954–0.97
Chronic bronchitis symptoms	Sensitivity	0.046	0.019–0.074	0.074	0.022–0.125
	Specificity	0.979	0.973–0.985	0.979	0.973–0.985
	LR+	2.244	1.161–4.337	3.520	1.664–7.443
	PPV	0.189	0.086–0.292	0.133	0.044–0.223
	NPV	0.908	0.896–0.920	0.960	0.952–0.968

ATS = American Thoracic Society; CI = confidence interval; ERS = European Respiratory Society; FEV_1_ = forced expiratory volume in 1 second; FEV_1_/FVC = FEV_1_/forced vital capacity ratio; Gold = Global Initiative for Chronic Obstructive Lung Disease; LR + = positive likelihood ratio; NPV = negative predictive value; PPV = positive predictive value; SD = standard deviation.

**Table 3:** Diagnostic accuracy of the question “Have you been diagnosed by a physician as having COPD or emphysema?” and of self-reported, questionnaire-based chronic bronchitis symptoms to detect chronic obstructive pulmonary disease (COPD) in men and women

			**FEV**_**1**_**/FVC <0.7 (GOLD)**		**FEV**_**1**_**/FVC <1.645 SD below predicted (ATS/ERS)**	
			**Value**	**95% CI**	**Value**	**95% CI**
Physician-diagnosed COPD	Sensitivity	Women	0.083	0.032–0.135	0.11	0.035–0.185
		Men	0.032	0–0.065	0.074	−0.015–0.163
	Specificity	Women	0.996	0.993–1	0.995	0.991–0.999
		Men	0.997	0.994–1	0.996	0.992–1
	PPV	Women	0.682	0.434–0.93	0.545	0.28–0.811
		Men	0.545	0.17–0.921	0.364	0.001–0.726
	NPV	Women	0.918	0.903–0.934	0.952	0.94–0.964
		Men	0.902	0.885–0.919	0.973	0.963–0.982
Chronic bronchitis symptoms	Sensitivity	Women	0.044	0.006–0.083	0.073	0.011–0.136
		Men	0.048	0.009–0.088	0.074	−0.015–0.163
	Specificity	Women	0.976	0.968–0.985	0.977	0.969–0.986
		Men	0.983	0.975–0.991	0.981	0.973–0.989
	PPV	Women	0.154	0.029–0.279	0.154	0.029–0.279
		Men	0.237	0.064–0.409	0.105	−0.019–0.23
	NPV	Women	0.914	0.898–0.929	0.949	0.937–0.962
		Men	0.902	0.885–0.92	0.972	0.963–0.982

ATS = American Thoracic Society; CI = confidence interval; ERS = European Respiratory Society; FEV_1_ = forced expiratory volume in 1 second; FEV_1_/FVC = FEV_1_/forced vital capacity ratio; Gold = Global Initiative for Chronic Obstructive Lung Disease; NPV = negative predictive value; PPV = positive predictive value.

**Table 4:** Diagnostic accuracy of the question “Have you been diagnosed by a physician as having COPD or emphysema?” to detect chronic obstructive pulmonary disease (COPD), in non-smokers, ex-smokers, and current smokers

		**FEV**_**1**_**/FVC <0.7 (GOLD)**		**FEV**_**1**_**/FVC <1.645 SD below predicted (ATS/ERS)**	
		**Value**	**95% CI**	**Value**	**95% CI**
Sensitivity	Non-smokers	0.009	−0.014–0.032	0.022	−0.033–0.077
	Ex-smokers	0.070	0.017–0.124	0.138	0.025–0.251
	Current smokers	0.087	0.021–0.153	0.117	0.013–0.220
Specificity	Non-smokers	1		1	
	Former smokers	0.992	0.986–0.998	0.991	0.985–0.997
	Current smokers	0.996	0.989–1.003	0.991	0.981–1.000
PPV	Non-smokers	1		1	
	Former smokers	0.5	0.22–0.78	0.4	0.126–0.674
	Current smokers	0.833	0.564–1.102	0.583	0.228–0.939
NPV	Non-smokers	0.941	0.928–0.955	0.976	0.967–0.985
	Former smokers	0.906	0.886–0.925	0.964	0.951–0.976
	Current smokers	0.830	0.792–0.867	0.914	0.886–0.942

ATS = American Thoracic Society; CI = confidence interval; ERS = European Respiratory Society; FEV_1_ = forced expiratory volume in 1 second; FEV_1_/FVC = FEV_1_/forced vital capacity ratio; Gold = Global Initiative for Chronic Obstructive Lung Disease; NPV = negative predictive value; PPV = positive predictive value.

**Table 5:** Diagnostic accuracy of self-reported, questionnaire-based chronic bronchitis symptoms to detect chronic obstructive pulmonary disease (COPD), in non-smokers, ex-smokers, and current smokers

		**FEV**_**1**_**/FVC <0.7 (GOLD)**		**FEV**_**1**_**/FVC <1.645 SD below predicted (ATS/ERS)**	
		**Value**	**95% CI**	**Value**	**95% CI**
Sensitivity	Non-smokers	0.044	−0.004–0.092	0.044	−0.032–0.121
	Ex-smokers	0.042	0–0.084	0.094	0.003–0.185
	Current smokers	0.052	0–0.104	0.067	−0.014–0.147
Specificity	Non-smokers	0.981	0.973–0.989	0.980	0.971–0.988
	Former smokers	0.979	0.968–0.989	0.980	0.970–0.990
	Current smokers	0.977	0.960–0.993	0.975	0.959–0.992
PPV	Non-smokers	0.128	−0.006–0.262	0.051	−0.037–0.14
	Former smokers	0.182	0.014–0.35	0.182	0.014–0.35
	Current smokers	0.333	0.056–0.611	0.222	−0.023–0.467
NPV	Non-smokers	0.943	0.929–0.956	0.976	0.967–0.985
	Former smokers	0.901	0.881–0.921	0.962	0.949–0.975
	Current smokers	0.821	0.783–0.86	0.908	0.879–0.937

ATS = American Thoracic Society; CI = confidence interval; ERS = European Respiratory Society; FEV_1_ = forced expiratory volume in 1 second; FEV_1_/FVC = FEV_1_/forced vital capacity ratio; Gold = Global Initiative for Chronic Obstructive Lung Disease; NPV = negative predictive value; PPV = positive predictive value.

## Discussion

### Diagnostic accuracy of the questionnaire

The question “Have you been diagnosed by a physician as having COPD or emphysema?” is commonly used in population-based epidemiological studies. In this general population-based study, it scored low sensitivity (5.7% for GOLD and 9.8% for LLN) and high specificity to detect significant airflow obstruction according to GOLD guidelines (99.7%) and ATS/ERS recommendations based on LLN (99.5%), as well as a rather high LR + and a reasonable PPV (63.6% and 48.5% for GOLD and LLN, respectively). These results suggest that the question is truly associated with COPD and prevents misclassification of non-COPD subjects by spirometry. Unfortunately, the low sensitivity implies that a significant number of subjects with COPD cannot be identified by this question, and reflects the well-known underdiagnosis of COPD by physicians [18], even using the more stringent LLN criteria [6]. However, in analytical epidemiological studies, especially when the aim is to evaluate a risk factor, it is preferable to have a test/question with very high specificity and lower sensitivity, to avoid false positive findings and, consequently, bias in risk estimates [19]. Analyzing a subsample of subjects >40 years old, at which age the disease is more likely to be diagnosed, did not significantly change the results.

The accuracy of questionnaire items to identify COPD has previously been criticized [20] and they will never replace spirometry to make diagnosis [21]. Unsurprisingly, the sensitivity in detecting COPD through asking the question “Have you been diagnosed by a physician as having COPD or emphysema?” is lower compared to that of the question “Have you been diagnosed by a physician as having asthma?” in defining true asthma cases [8] because asthma is more widely known to patients and physicians. Recently, the accuracy of questionnaire items in defining COPD was assessed with the main purpose of developing a screening tool for primary care [11,22]; in these studies, a set of mixed questions on symptoms and personal information achieved good sensitivity and reasonable specificity. However, they included only high risk groups (smokers), excluding those with a self-reported doctor diagnosis of COPD, making comparison impossible. In another study, female nurses reporting a physician diagnosis of COPD were selected to perform pulmonary function tests. The results showed a slightly higher accuracy than seen in our population [13]. This could possibly be explained by the sample selection. Health care professionals, because of their education, are probably more likely to correctly report their medical history compared to non-health care professionals. Moreover, since women are less likely than men with an identical medical history to receive a physician diagnosis of COPD [23], women have a higher probability of having COPD when reporting physician-diagnosed COPD, as also shown in our data (Table 3). Not surprisingly, the question about COPD was more accurate in indicating airway obstruction compared to the combination of questions that defined chronic bronchitis (cough and phlegm for at least 3 months, 2 years consecutively). This suggests that subjects other than persons with COPD (e.g., subjects with asthma or repeated respiratory infections) also reported these symptoms. In fact, subjects with chronic bronchitis have a higher rate of respiratory infections (40%) compared to subjects with COPD (33.1%) and the general population (33.2%) (data not shown). Surprisingly, just 6.1% of the participants with a history of physician-diagnosed COPD had chronic bronchitis symptoms, suggesting that COPD could be underdiagnosed or misdiagnosed. Given the high number of subjects with wheeze (24.6% using the GOLD definition and 31.3% with the LLN definition), we cannot exclude that asthma was also underdiagnosed or underreported in our population. However, wheezing is often also associated with COPD [24] and the results did not change significantly when the analysis was performed in the subjects not reporting wheezing (data nor shown).

The accuracy of questionnaire questions in detecting airflow obstruction did not differ between men and women. The reported question on doctor-diagnosed COPD showed higher sensitivity in detecting COPD in smokers compared to non-smokers, whereas the PPV was higher in current smokers than in non-smokers. Just one non-smoker reported doctor-diagnosed COPD, but in non-smokers there was no false positive result. This interaction of smoking habits with the accuracy of the question is not unexpected and could be related to the well-known underdiagnosis of COPD in primary care, where physicians are more likely to make the diagnosis if the patient is a smoker [25].

In the present study, chronic bronchitis symptoms were fairly common in those who were excluded because of reporting physician-diagnosed asthma (8.6%), higher than in subjects with COPD according to either the GOLD or the LLN definition (data not shown).

### Validity issues

In this study, only pre-bronchodilator spirometric data was available. Because the GOLD guidelines give a FEV_1_/FVC ratio of <0.7 as cutoff point to diagnose COPD, based on post-bronchodilator data, and the American College of Chest Physicians, American College of Physicians, ATS, and ERS define COPD as a disease characterized by an airflow obstruction not fully reversible [3], this could be considered the main limitation of this study, bearing to an overdiagnosis of COPD. Anyway, the prevalence of COPD in our sample of the general population was lower than expected, based on previous studies [26,27], even if the FEV_1_/FVC ratio was derived from pre-bronchodilator data. Nonetheless, in another, similar study, a pre-bronchodilator FEV_1_/FVC ratio was accepted [28] and at the time the present study was performed the guideline of the UK National Institute for Clinical Excellence did not consider spirometric reversibility testing a necessary part of the diagnostic process [29]. As a matter of fact, the bronchodilator response suffers from a lack of reproducibility [30,31], being influenced by smoking habits and other parameters [32], and failed to discriminate between asthma and COPD [31,33]. However is undeniable that pharmacological bronchodilation with salbutamol would influence FEV1/FVC ratio, increasing it in normal subjects and in patients with mild stages of COPD [34]. This expected increase could affect sensitivity and other measures of validity in this study. Unfortunately the variability of post-bronchodilator response in FEV1/FVC found in other studies [34,35] and the lack of data on post-bronchodilator response in our study makes very difficult to estimate this effect in our population.

The choice of using the European Community for Steel and Coal (ECSC)/ERS equation [15] to calculate LLN could be another limitation, since it will not take in consideration the non-linear decline of FEV1 related to the age. Nevertheless the aim of this study was to assess the diagnostic accuracy of a questionnaire and, to make our results comparable and applicable in clinical and epidemiological practice, we had to rely on those methods still most widely used, despite some relevant limitations.

The Finally, the subjects we excluded because they did not respond to the questions regarding smoking could have biased our accuracy estimates. However, a further analysis including these subjects confirmed our results (data not shown).

## Conclusions

This was the first study to assess the accuracy in detecting COPD through a frequently used questionnaire item. The high specificity and good PPV suggest that the question “Have you been diagnosed by a physician as having COPD or emphysema?” is more likely to identify those who do not have the disease, whereas the low sensitivity of this question could underestimate the real burden of COPD in the general population.

## Competing interest

All authors declared to *COPD* that no potential conflicts of interest exist with any companies/organizations whose products or services may be discussed in this article.

This study was supported by grants from the Västra Götaland County Council, the Swedish Council for Working Life and Social Research, the Swedish Research Council, the Swedish Heart and Lung Foundation, and the Swedish Research Council for Environment, Agricultural Sciences and Spatial Planning.

## Authors’ contributions

KT, ACO and NM designed the study. AC, KT, JB were responsible for data collection. NM, KT, and GM managed and analysed the data. All authors participated in the interpretation and final drafting of the manuscript. All authors read and approved the final manuscript.

## Pre-publication history

The pre-publication history for this paper can be accessed here:

http://www.biomedcentral.com/1471-2466/14/49/prepub
